# Predictors of Spontaneous Remission of Problematic Internet Use in Adolescence: A One-Year Follow-Up Study

**DOI:** 10.3390/ijerph17020448

**Published:** 2020-01-09

**Authors:** Lutz Wartberg, Katajun Lindenberg

**Affiliations:** 1 Department Psychology, Faculty of Life Sciences, MSH Medical School Hamburg, 20457 Hamburg, Germany; 2 Institute for Psychology, University of Education Heidelberg, 69120 Heidelberg, Germany

**Keywords:** Internet addiction, pathological Internet use, Internet gaming disorder, gaming disorder, adolescent, remission, emotion regulation, self-efficacy, psychopathology, longitudinal study

## Abstract

Problematic use of the Internet is becoming increasingly important and especially for adolescents, high prevalence rates are reported in many countries. Despite the growing international research activities and the reported prevalence estimates, comparatively very few studies have focused on spontaneous remission and its possible causes. In a risk population of 272 adolescents, we used standardized diagnostic instruments to investigate which socio-demographic and psychosocial characteristics at baseline (at t1) predicted spontaneous remission of problematic Internet use one year later (at t2). The predictors were determined by bivariate and multivariate logistic regression analyses. In the bivariate regressions, we found male gender, higher self-efficacy (t1), a lower level of maladaptive emotion regulation strategies (t1), lower depression (t1), lower performance and school anxiety (t1), lower social-interaction anxiety (t1), and lower procrastination (t1) to predict spontaneous remission of problematic Internet use at t2. In the multivariable analysis, a lower level of maladaptive emotion regulation strategies (t1) was the sole statistically significant predictor for the remission one year later (t2). For the first time, the high relevance of emotion regulation for spontaneous remission of adolescent problematic Internet use was observed. Based on these findings, emotion regulation could be specifically trained and promoted in future prevention measures.

## 1. Introduction

In adolescence, the use of different Internet applications is widespread all over the world. This applies to both for school settings (e.g., information search) as well as for leisure activities (e.g., communication with messengers, watching videos, or playing computer games). Among many positive aspects, negative effects of excessive Internet use were observed, especially in adolescents (e.g., see the comprehensive overview by Cerniglia et al. [1]). According to the definition of Shaw and Black [2], problematic Internet use (often also named as Internet addiction) is “… characterized by excessive or poorly controlled preoccupations, urges or behaviours regarding computer use and internet access that lead to impairment or distress” (p. 353). Research activities and the number of scientific publications on problematic Internet use have grown rapidly over the last few years [3]. However, the emergence of problematic Internet use in youth has not yet been clearly clarified, and there are also very few empirical findings on the stability or spontaneous remission of this behavior pattern over time (e.g., Lau et al. [4]).

In his cognitive–behavioral model for the development of problematic Internet use, Davis [5] makes a distinction between “generalized pathological Internet use” and “specific pathological Internet use” (concerning the problematic use of single applications on the Internet, such as online games). Only the latter form has found its way into the international classification systems so far. Problematic use of video games was incorporated as “Internet Gaming Disorder” in the DSM-5 [6] and as “Gaming Disorder” in the ICD-11 (e.g., [7]). In contrast, the inclusion of a generalized or general problematic use of the Internet in the classification systems has been intensively discussed [8] but has not yet taken place. Up to now, there is still no international consensus on uniform criteria for the diagnosis of a general problematic Internet use (e.g., [9]). Furthermore, the concept of problematic Internet use is discussed quite controversially, for example because it pertains to a variety of very different online behaviors [10] or it is seen as compensatory behavior [11]. As a current alternative to existing models (e.g., [5]), Kardefelt-Winther [11] developed the compensatory Internet use theory (CIUT), which could also be used as a basis for research on problematic Internet use. Starcevic and Aboujaoude [10], who have carried out a comparison of different screening tools for problematic Internet use, reported as most frequently used diagnostic criteria (A) withdrawal symptoms, (B) development of tolerance, and (C) negative consequences of Internet use.

Meanwhile, in adolescence the prevalence of a general problematic Internet use has been investigated in various international surveys. In Europe and North America, prevalence estimates for adolescents were generally lower than in Asia [12], where prevalence rates up to 26.7% were reported [13]. In Germany, where this study was conducted, prevalence estimates for problematic Internet use in representative samples of adolescents ranged from 3.2% to 4.7% [14,15,16]. For single specific Internet applications, a representative survey of German youth revealed prevalence estimates of 3.5% for problematic video game use and 2.6% for problematic social media use [17]. Accordingly, these problematic behavior patterns seem to affect a substantial proportion of adolescents. Moreover, problematic Internet use seems to be associated with psychosocial impairment, for example lower self-efficacy [18], more difficulties in emotion regulation [19], psychopathological burden as stronger conduct problems and depressive symptoms [20] or social anxiety [21], but as well as with procrastination [22] and learning [23]. In contrast to the intensive international research activities in this field and the reported prevalence rates, comparatively very few studies have focused on spontaneous remission and its possible causes.

With regard to the stability of symptoms, Strittmatter et al. [24] found persistent problematic Internet use over two years in 0.58% of German adolescents. This frequency, however, needs to be interpreted in context of the prevalence estimates for Germany (between 3% and 5%) in adolescence [14,15,16]. In a further study, a stable problematic Internet use over a period of two years was found in 4.8% of investigated youth, and a corresponding kappa coefficient of 0.27 was observed [25].

There are currently very few findings on the spontaneous remission of problematic Internet use in adolescence. Ko et al. [26] found low hostility and low interpersonal sensitivity as predictors for a remission of problematic Internet use among Taiwanese youth over one year. In another survey, Ko et al. [27] examined adolescents in Taiwan over a period of one year and reported that “depression, hostility, and social anxiety decreased in the process of remission” (p. 1377). In a sample of Chinese secondary school students, Lau et al. [4] observed various different predictors for a remission of problematic Internet use over a period of one year. Concerning psychosocial characteristics at the time of the first measurement, they obtained as predictors higher self-esteem, lower social anxiety, and lower severe depression, whereas over time changes in depression, anxiety, loneliness, self-esteem, positive affect, and family support were identified [4]. In summary, the findings regarding the remission of problematic Internet use in adolescence are currently still very limited. Only a few predictors could be replicated in the published studies, these were lower social anxiety and depression [4,27] as well as lower hostility [26,27].

The aim of the present analysis was to examine predictors of spontaneous remission of problematic Internet use among German adolescents. We explored the following research question: What socio-demographic and psychosocial characteristics in adolescence can predict spontaneous remission (without treatment) of problematic Internet use within one year?

## 2. Materials and Methods

### 2.1. Procedure

The data collection took place within the framework of the PROTECT study (ClinicalTrials.gov: NCT02907658). The aim of the PROTECT study was to investigate the effectiveness of a school-based preventive measure against Internet use disorders in a randomized controlled design (but in this analysis on spontaneous remission of adolescent problematic Internet use, only the data of the untreated control group were utilized). The ethics vote for the PROTECT study was obtained by the Research Ethics Committee of the University of Education Heidelberg (Az.: 7741.35-13) as well as the consent of the Regional Council (Az.: 71c2-6499.25). An informed written consent was obtained both from the adolescents interviewed and from one of their parents. Data were collected between September 2015 and February 2018 by trained psychologists in a total of 34 schools in the three federal states of Baden-Württemberg, Hesse and Rhineland-Palatinate during regular school hours. For the present investigation, the data of the first measurement (t1) were used as predictors for the data 12 months later (t2). For the PROTECT study, a risk population was recruited, including only youth who achieved a sum value of at least 20 in the applied screening instrument for problematic Internet use (Compulsive Internet Use Scale, CIUS [28]). Since a total value of ≥24 points is an empirical basis for assuming problematic Internet use [29], these are adolescents with a higher risk of but not necessarily with a problematic pattern of use. Furthermore, the study participants were not allowed to be in treatment because of their use of the Internet or computer games at t1. A detailed description of the sampling procedure can be found in the study protocol of the PROTECT study [30].

### 2.2. Measures

In order to assess a problematic Internet use, the German-language version [31] of the Compulsive Internet Use Scale [28] was applied at t1 and at t2. The CIUS consists of 14 items with a 5-level response format (0 = “never” to 4 = “very often”). The total score (range: 0 to 56 points) is used to define symptom severity. In the present sample, we observed reliability coefficients (Cronbach’s *α*) for the CIUS of 0.54 (t1) and 0.83 (t2). According to the findings of Bischof et al. [29], a sum score of ≥24 points indicates a problematic Internet use. Bischof et al. [29] used standardized, fully structured diagnostic interviews and determined an empirical cut-off value for the CIUS based on the interview results. They recommend as an empirically determined cut-off value for the CIUS a “…threshold of 24 as appropriate…” ([29] p. 54).

Self-efficacy at t1 was assessed with the General Self-Efficacy Scale (Skala zur Allgemeinen Selbstwirksamkeitserwartung, SWE) [32]. The SWE comprises 10 items with a 4-level response format (1 = “not at all true” to 4 = “exactly true”). Higher values of the sum score indicate a higher expectation of self-efficacy. In the present sample, Cronbach’s *α* for the SWE was 0.87.

Adaptive and maladaptive strategies of habitual emotion regulation at t1 were assessed with the Questionnaire for Assessment of Emotion Regulation in Children and Adolescents (Fragebogen zur Erhebung der Emotionsregulation bei Kindern und Jugendlichen, FEEL-KJ; [33]) regarding the two emotions anxiety and sadness with a total of 60 items. The answer format for all FEEL-KJ questions is five-level (1 = “almost never” to 5 = “almost always”). For each emotion, 15 emotion regulation strategies (subscales or first-order scales) can be calculated. Seven of these subscales (“problem-oriented action”, “distraction”, “lifting mood”, “acceptance”, “forgetting”, “cognitive problem solving”, and “reevaluation”) are summarized as second-order scales of “adaptive emotion regulation strategies”. Further five scales (“giving up”, “aggressive behavior”, “withdrawal”, “self-devaluation”, and “perseveration”) are summarized to the second-order scale “maladaptive emotion regulation strategies”. The remaining subscales can be assigned to a third second-order scale (“other strategies”), which, however, was not used in the present analysis. The two second-order scales adaptive and maladaptive emotion regulation strategies were summarized for the two emotions anxiety and sadness by summing up the two mean values and higher total values indicated for stronger pronounced strategies. In the present sample, Cronbach’s *α* for adaptive emotion regulation strategies was 0.91 and for maladaptive emotion regulation strategies 0.88.

General psychopathology at t1 was calculated with the total score of the Strengths and Difficulties Questionnaire (SDQ) [34]. The SQD consists of 25 items with a 3-level response format (0 = “not true” to 2 = “certainly true”). Based on the 25 questions, five scales (“emotional symptoms”, “conduct problems”, “hyperactivity/inattention”, “peer (relationship) problems”, and “prosocial behavior”) can be calculated. The sum score of the SDQ was determined from the first four (problem) scales. Higher values indicate higher psychopathology. In the present sample, Cronbach’s *α* for the SDQ total score was 0.68.

Depressive symptoms at t1 were assessed using the German version of the Children’s Depression Inventory (DIKJ) [35]. The DIKJ comprises 26 items, each with three predefined answer alternatives (scored 0 to 2). The sum score is used to indicate the symptom severity, whereas higher values stand for higher levels of depressiveness. In the present sample, Cronbach’s *α* for the DIKJ total score was 0.85.

Performance and school anxiety at t1 were assessed with a total of nine questions from the German version of the Fear Survey Schedule for Children—Revised (Phobiefragebogen für Kinder und Jugendliche, PHOKI) [36]. The PHOKI items have a three-level response format (0 = “not at all” to 2 = “often”). Based on the nine answers, a sum score was calculated, and a higher value indicates a stronger symptom severity. In the present sample, Cronbach’s *α* for the PHOKI items was 0.76.

Social anxiety at t1 was assessed with the German version of the Social Interaction Anxiety Scale (SIAS) [37]. The SIAS consists of 20 items with a five-level answer format (0 = “not applicable at all” to 4 = “very strongly applicable”). Higher sum scores indicate a stronger expression of fear in social interactions. In the present sample, Cronbach’s *α* for the SIAS was 0.89.

Procrastination, the tendency to postpone unpleasant tasks, was investigated at t1 with the Questionnaire for Procrastination (Allgemeiner Prokrastinationsfragebogen, APROF) [38]. The APROF comprises a total of 18 items with a seven-level response format (1 = “never” to 7 = “always”). The answers are summed up, and a higher total score stands for a stronger tendency to procrastinate. In the present sample, Cronbach’s *α* for the APROF was 0.94.

The social and learning behavior at t1 was assessed with the Student Assessment List for Social and Learning Behavior (Schülereinschätzliste für Sozial- und Lernverhalten, SSL) [39]. The SSL consists of 40 items with a four-level response format (0 = “never” to 3 = “often”) from which a total of 10 scales can be formed. The six subscales “cooperation”, “self-perception”, “self-control”, “empathy”, “adequate self-assertion”, and “social contact” were combined to the (second-order) scale “social behavior” (Cronbach’s *α* = 0.77). The (second-order) scale “learning behavior” was determined from the four scales “perseverance/willingness to work hard”, “concentration”, “independence in learning”, and “diligence in learning” (Cronbach’s *α* = 0.77). Higher values represent a more pronounced social/learning behavior.

### 2.3. Statistical Analyses

As previously mentioned, spontaneous remission of a problematic Internet use was considered exclusively for the participants of the untreated control group (*n* = 272) of the PROTECT study. In the PROTECT study, the control group remained untreated and therefore did not receive any intervention during the course of the examination (therefore remission cannot be attributed to a preventive or therapeutic measure). Spontaneous remission was defined by (a) problematic Internet use (CIUS ≥24) at t1 and (b) unproblematic Internet use (CIUS <24) at t2. Thus, the inclusion criterion to be considered for the analysis was to meet criterion (a). Then, in the analysis, the groups of affected adolescents who also met criterion (b) one year later (=spontaneous remission) versus those who did not meet criterion b) (=persistent pathology) were compared.

Finally, 134 adolescents with a CIUS score ≥24 at t1 were included in the analysis. The subjects who had a CIUS sum value of <24 at t2 (*n* = 96) were assigned to the group with spontaneous remission of problematic Internet use. Those adolescents whose CIUS sum value was ≥24 at t2 (*n* = 38) were allocated to the group with persistent problematic Internet use. Frequencies, mean values, standard deviations, and reliability coefficients were calculated. Predictors for a spontaneous remission of problematic Internet use were determined by binary logistic regression analyses. In all calculated analyses, the dependent variable in the regression model was the group affiliation at t2 (coding: “spontaneous remission of problematic Internet use” = 1 versus “persistent problematic Internet use” = 0). Gender, age, self-efficacy, adaptive emotion regulation strategies, maladaptive emotion regulation strategies, general psychopathology, depression, performance and school anxiety, social-interaction anxiety, procrastination, social behavior, and learning behavior (each collected at t1) were used as independent variables. In the first step, the independent variables were included into bivariate logistic regression models and then, in the second step, simultaneously included in a multivariate regression model. All statistical evaluations were carried out with the statistical software SPSS version 25.0 (IBM, 2017, New York, NY, USA).

## 3. Results

### 3.1. Descriptive Statistics

For the included 134 adolescents (all with a CIUS sum score of ≥24 points at t1), a detailed description of the sample characteristics at the first measurement time (separately for the two groups with and without remission of problematic Internet use at t2) can be found in Table 1. A total of 7.5% of the sample attended school at a low educational level, 17.9% at medium educational level, and 74.6% at high educational level.

### 3.2. Bivariate Logistic Regression Analyses

In the bivariate regressions, we found male gender, higher self-efficacy (t1), lower levels of maladaptive emotion regulation strategies (t1), lower depression (t1), lower performance and school anxiety (t1), lower social-interaction anxiety (t1), and lower procrastination (t1) to predict spontaneous remission of problematic Internet use at t2 one year later (see Table 2).

### 3.3. Multivariable Logistic Regression Analysis

In the multivariable analysis, only lower levels of maladaptive emotion regulation strategies (t1) predicted the spontaneous remission of problematic Internet use one year later (t2).

## 4. Discussion

In this longitudinal study, we examined socio-demographic and psychosocial predictors of spontaneous remission of problematic Internet use in adolescence. So far there have been very few published findings regarding spontaneous remission: Two studies conducted in Taiwan [26,27] and one study from China [4]. The results of the present study and the three published surveys showed both similarities and differences. The most important finding of this study is the fact that a spontaneous remission of problematic Internet use within 12 months is strongly predicted by less pronounced maladaptive emotion regulation strategies. In the multivariate model, less pronounced maladaptive strategies of emotion regulation proved to be the only statistically significant predictor for the remission of a juvenile problematic Internet use. The maladaptive strategies were already the strongest predictor in the bivariate logistic regressions (with the highest explained variation between the two groups with and without remission of problematic Internet use), and this finding was again shown in the multivariable model. Since emotion regulation was not investigated directly in the three published surveys on remission of problematic Internet use [4,26,27], it is essential to analyze the role of maladaptive emotion regulation in the theoretical frameworks of depression. Clinical etiology models of depression, such as the “behavioral approach to depression” [40], the theory of learned helplessness [41], and Beck’s cognitive theory of depression [42] emphasize the role of maladaptive emotion regulation in the emergence and maintenance of depression, e.g., the maladaptive emotion regulation strategy withdrawn is associated with a loss of positive reinforcement, which has been found to be a significant cause and maintenance factor of depression [40]. In line with the theory of learned helplessness [41], giving up is another maladaptive emotion regulation strategy that is associated with the maintenance of depression because it reduces the motivation to change. Moreover, the cognitive theory of depression according to Beck [42] emphasizes the process of devaluing oneself, the future and others as a significant maintenance factor of depression [42].

The results by Lau et al. [4] and Ko et al. [27] showed in particular the importance of depression for the remission of problematic Internet use. In several previous surveys, relations between problematic Internet use and higher severity of depressive symptoms were observed (e.g., [20]). The important role of depression was also demonstrated in the study of Chang et al. [43]. Chang et al. [43] examined the associations from the opposite perspective and stated that compared to a group with remission in the group with stable problematic behavior over one year “an increase in the existence of depression and alcohol use...” “...predicted the persistence of Internet addiction” (p. 1434). In the present study, the bivariate analysis also showed a relation between lower depression and spontaneous remission, but this finding was not retained in the multivariate analysis (see Table 2). One possible explanation is that it is not the depression itself but the maladaptive emotion regulation strategies associated with depression that lead to the problematic Internet usage behavior.

A similar picture emerged with regard to social anxiety. Associations between problematic Internet use and social anxiety were found earlier in some published studies (e.g., [21]). The relevance of social anxiety for the spontaneous remission of adolescent problematic Internet use was already reported before by Lau et al. [4] and Ko et al. [27]. In the present study, social-interaction anxiety was a predictor for spontaneous remission in the bivariate regression, but again this finding could not be confirmed in multivariate analysis. Further research seems necessary to clarify whether depression and social anxiety can be replicated as predictors for the spontaneous remission of problematic Internet use or (as the findings of the present study suggest) emotion regulation may be the even more important mechanism underlying both depression and social anxiety. Further interesting potential predictors for future research projects are self-esteem [4], hostility [26,27], and according to the bivariate findings of this study, lower performance and school anxiety and lower procrastination.

The bivariate finding in the present study that boys show spontaneous remission more frequently than girls is also interesting. Of course, this result also needs to be confirmed in future studies but would then be quite important, since in Germany girls and boys are affected by a similar frequency of problematic Internet use (e.g., [16,44]). If the probability of spontaneous remission is actually higher in male adolescents, it might be helpful to develop prevention or intervention measures that are gender-specific (especially, interventions for affected girls should not be neglected).

The present study shows various limitations. First and foremost, at t1 the reliability of the CIUS was surprisingly low (perhaps due to the rather homogeneous risk sample of students, because reliability is always a property of a measurement and in a very homogeneous sample reliability may be lower than in a heterogeneous sample) [45], whereas the reliability coefficient for the CIUS at t2 was satisfactory. In previous studies, good reliability coefficients were observed for the German version of the CIUS (e.g., [31]). However, it cannot be ruled out that, for example, changes in general Internet usage behavior over time will be less well described by the item formulations and thus make the questionnaire less reliable (this potential problem of the CIUS should be examined in future studies). In the interpretation of the findings, it should be taken into account that the lower reliability at t1 leads to a larger confidence interval or a less precise measurement of problematic Internet use [45]. Due to economic reasons, not all relevant constructs could be included. For example, the characteristic hostility, whose relevance was shown in the both studies of Ko et al. [26,27], was not surveyed in this examination. In the present study, a risk sample was investigated, so it remains unclear to what extent the findings can be generalized for the general population. Alternatively, in the case of prevalence rates of between three and five percent for problematic Internet use in adolescence in Germany [14,15,16], very high case numbers are required in a general population sample in order to be able to investigate a sufficient number of adolescents with regard to spontaneous remission over time. In general, the incidence, the new occurrence of problematic Internet use within the period of a longitudinal study, is also interesting and has also been explored in other studies [26]. However, this phenomenon was found in very few adolescents in the present study, so the low case numbers for incidence (in contrast to spontaneous remission) did not provide a sufficient basis for statistical analyses.

## 5. Conclusions

The findings of the present study supplement the currently still very limited state of research on factors that influence the spontaneous remission of problematic Internet use in adolescence. For the first time the great importance of maladaptive emotion regulation strategies as a significant maintenance factor of problematic Internet use could be observed. This is of high relevance for research and practice. Should this result be confirmed in further studies, it would be a promising approach to specifically address the regulation of emotions in youth (e.g., in the context of prevention measures). In fact, an early intervention study has already shown that targeted training with a focus on improving emotion regulation strategies significantly improves the core symptoms of problematic Internet use over four months [46]. A targeted positive development of emotion regulation in childhood and adolescence (e.g., reduction of maladaptive strategies in life competence trainings) could then contribute to promote spontaneous remission regarding problematic Internet use. This could possibly on the one hand reduce the number of affected adolescents and on the other hand save costs and resources that could otherwise be incurred for follow-up treatment in a relatively economic way (life competence trainings are often used in school settings in Germany anyway).

## Figures and Tables

**Table 1:** Socio-demographic and psychosocial characteristics of adolescents with and without spontaneous remission of problematic Internet use at t1.

Variable	Remission of Problematic Internet Use (t2) % or *M* (Standard Deviation; SD)	No Remission of Problematic Internet Use (t2) % or *M* (SD)
Gender	51.0% (girls); 49.0% (boys)	73.7% (girls); 26.3% (boys)
Age (t1)	15.04 (1.84)	15.21 (1.89)
Problematic Internet use (t1)	28.80 (4.64)	31.40 (6.10)
Self-efficacy (t1)	27.42 (4.35)	25.40 (5.23)
Adaptive emotion regulation strategies (t1)	6.22 (1.19)	6.12 (1.09)
Maladaptive emotion regulation strategies (t1)	5.35 (1.32)	6.46 (1.23)
General psychopathology (t1)	12.45 (4.70)	14.21 (4.94)
Depression (t1)	14.62 (6.38)	19.12 (8.09)
Performance and school anxiety (t1)	6.50 (3.31)	8.35 (4.17)
Social-interaction anxiety (t1)	25.01 (11.92)	31.55 (15.04)
Procrastination (t1)	69.42 (20.93)	79.48 (19.24)
Social behavior (t1)	56.52 (9.19)	54.61 (11.13)
Learning behavior (t1)	35.57 (7.67)	33.97 (7.93)

**Table 2:** Bivariate and multivariable logistic regression analyses concerning predictors of spontaneous remission of problematic Internet use.

Variable	Remission of Problematic Internet Use (t2) Bivariate Regression Models Unadjusted Odds Ratios (95% Confidence Interval; CI)	Remission of Problematic Internet Use (t2) Multivariable Regression Model Adjusted Odds Ratios (95% CI)
Gender ^a^	0.37 * (0.16; 0.85)	0.53 (0.18; 1.55)
Age (t1)	0.95 (0.78; 1.16)	1.04 (0.81; 1.34)
Self-efficacy (t1)	1.10* (1.01; 1.19)	0.98 (0.87; 1.10)
Adaptive emotion regulation strategies (t1)	1.08 (0.78; 1.51)	0.83 (0.54; 1.28)
Maladaptive emotion regulation strategies (t1)	0.53 *** (0.39; 0.73)	0.54 ** (0.34; 0.84)
General psychopathology (t1)	0.93 (0.86; 1.00)	1.14 (0.97; 1.32)
Depression (t1)	0.92 ** (0.87; 0.97)	0.94 (0.85; 1.05)
Performance and school anxiety (t1)	0.87 * (0.78; 0.97)	0.88 (0.76; 1.02)
Social-interaction anxiety (t1)	0.96 * (0.94; 0.99)	1.00 (0.95; 1.04)
Procrastination (t1)	0.98 * (0.96; 1.00)	1.00 (0.97; 1.02)
Social behavior (t1)	1.02 (0.98; 1.06)	1.02 (0.97; 1.08)
Learning behavior (t1)	1.03 (0.98; 1.08)	1.01 (0.94; 1.09)
Nagelkerke’s *R*^2^	–	0.29

Note. ^a^ Coding: 0 = male, 1 = female. * *p* < 0.05; ** *p* < 0.01; *** *p* < 0.001.
